# The Development of the Tobacco Tactics Website

**DOI:** 10.2196/resprot.2445

**Published:** 2013-06-28

**Authors:** Sonia A Duffy, Karen E Fowler, Petra S Flanagan, David L Ronis, Lee A Ewing, Andrea H Waltje

**Affiliations:** ^1^Veterans Affairs Ann Arbor Healthcare SystemCenter for Clinical Management ResearchAnn Arbor, MIUnited States; ^2^University of MichiganSchool of NursingAnn Arbor, MIUnited States; ^3^University of MichiganDepartments of Otolaryngology and PsychiatryAnn Arbor, MIUnited States; ^4^Veterans Affairs Ann Arbor Healthcare SystemAnn Arbor, MIUnited States

**Keywords:** smoking cessation, Internet, intervention studies, health services accessibility

## Abstract

**Background:**

Web-based cessation interventions have been shown to reduce tobacco use, be more efficacious than self-help booklets, be more efficacious if they provide tailored messages, and enhance quit rates in conjunction with nicotine replacement therapy.

**Objective:**

The objective of this study was to usability test and pilot test the Tobacco Tactics website for veterans.

**Methods:**

Both formative and summative evaluations were used across three small successive studies to develop and test the Tobacco Tactics website for veterans, which was based on a prior face-to-face smoking cessation intervention. Once the website was developed, the research team and Web developers usability tested the website with 5 veteran smokers and former smokers. Feedback from the veterans was collected as they navigated each webpage, then used to revise the website. In pilot study 1, 9 veteran smokers were provided access to the website, and given a baseline and 30-day follow-up survey. In pilot study 2, 18 veteran smokers, who were also motivated to quit smoking, were recruited and randomized to either the Tobacco Tactics website plus nicotine replacement therapy or to the 1-800-QUIT-NOW telephone line.

**Results:**

As a result of usability testing, more than 27 modifications were made to improve the website. In pilot study 1, 50% (3/6) veterans who entered the website had cut down on the number of cigarettes and 83% (5/6) found the website enjoyable, easy to read, easy to navigate, and would recommend the website to others. In pilot study 2, which included only smokers motivated to quit and also offered nicotine replacement therapy, seven-day point prevalence abstinence at 30-day follow-up was 40% (4/10) in the intervention group compared to 13% (1/8) in the control group.

**Conclusions:**

These preliminary results are promising and suggest the need for wider-scale testing of the Tobacco Tactics website for veterans.

## Introduction

### Smoking in the Department of Veterans Affairs System

While smoking rates among veterans have decreased from 33% in 2001 to 22.2% in 2005, smoking remains especially pronounced among subgroups of veterans, such as returning Operation Enduring Freedom/Operation Iraqi Freedom (OEF/OIF) veterans [1,2]. Compared to nonsmokers, smokers have twice as many hospital stays and longer hospitals stays, resulting in greater hospital expenses per admission [3]. Treatment of heart attacks and strokes is expensive, especially in the Department of Veterans Affairs (VA) system where patients have less social support and economic resources. Studies have shown that veterans are interested in quitting, but those who are inpatients, older, not married, unemployed, and living in rural areas are less likely to receive cessation services [4,5]. In fact, less than 7% of smokers in the VA receive smoking cessation pharmaceuticals and cessation medications—which accounts for less than 1% of the VA pharmacy budget [6]—yet smoking-related illnesses may account for up to 21% of all VA health care costs [7].

Within the VA, outpatient programs provide the majority of services to smokers, in accordance with the Agency for Healthcare Research and Quality (AHRQ) guidelines [8], yet outpatient smoking cessation programs are poorly attended and few smokers are reached [9]. More recently, VA primary care providers have been more aggressive in providing cessation interventions in primary care clinics and inpatient units, but factors such as provider time, training, and competing demands limit their effectiveness [10]. The Committee on Smoking Cessation in Military and Veteran Populations and the Institute of Medicine recommended the development of a VA smoking cessation website for veterans [11]. VA cessation providers met in Atlanta in December 2007 and one recommendation was to develop a cessation website for veterans [12]. Since the face-to-face Tobacco Tactics intervention was found to be efficacious in a clinical trial [13], the intervention was developed into a website for smokers and usability tested and pilot tested among veterans.

### Theoretical Framework

Social marketing theory, which was used to design and refine the Tobacco Tactics website, is the planning and implementation of programs designed to bring about social change. Social marketing uses concepts from commercial marketing including the “4 Ps”: (1) create an enticing “Product” (ie, the package of benefits associated with the desired action such as the tailored Tobacco Tactics website), (2) minimize the “Price” the target audience believes it must pay in the exchange, such as easy access to the website and cessation medications, (3) make the exchange available in “Places” that reach the audience and fit its lifestyles, such as accessing the website from home or public libraries, and (4) “Promote” the exchange opportunity with creativity and through channels and tactics that maximize desired responses such as a tailored, multimedia, interactive website enhanced with medications and follow-up [14]. A central component of social marketing is listening to the needs and desires of the target audience and building the program based on their feedback. In social marketing, it is also important to evaluate the competition or rival offerings, such as competing cessation programs.

### Studies Leading to the Development of the Tobacco Tactics Website (Product)

The Tobacco Tactics website was based on the face-to-face Tobacco Tactics intervention (VA IIR 98-500), which was tested among head and neck cancer patients and showed a significant difference in 6-month smoking cessation rates when compared to an enhanced usual care group [13]. Having packaged the Tobacco Tactics intervention into a toolkit, a VA implementation study (VA SDP 06-003) [15] brought the face-to-face intervention to veteran inpatient smokers and found a significant improvement in 6-month quit rates. Social marketing techniques were used to engage veterans to assist in the development of the image-based VA Tobacco Tactics program logo and campaign character (VA Public Health Grant) [16].

### Efficacy of Internet Cessation Interventions (Product)

Web-based cessation interventions have been shown to reduce tobacco use [17-19], be more efficacious than self-help booklets [20], be more efficacious if they provide tailored messages [21], and enhance quit rates in conjunction with nicotine replacement therapy (NRT) [20-22]. A recent 2009 meta-analysis [23] reviewing 22 Web-based randomized control trials for smoking cessation yielded an abstinence rate about 1.5 times higher than the control group (intervention group 9.9%, control group 5.7%). Web-based cessation interventions can be further enhanced if they include tailored messages [21] and medications such as NRT [20-22].

### Advantages of Web-Based Interventions (Price/Place)

The Internet is a valuable tool for providing health education and support given its 24-hour accessibility, growing number of Internet users, potential to reach thousands of individuals, and potential cost-effectiveness [24]. The computer’s availability, enormous memory, versatile means of expression, and low cost make it a valuable adjunct in patient education and support [25]. *Tailored modules* are a superior alternative to “surfing the net” [22,24,26] because the message is adapted to the prior knowledge and characteristics of each individual, and hence contains more relevant information that increases attentiveness and limits defensiveness toward the intervention content [27]. *Interactivity* allows patient tailoring, increases engagement in decision making, improves learning, increases program attractiveness, and enhances the influence of online services [28]. *Multimedia* programming refers to “text, graphics, audio and video with links and tools that allow the user to navigate, interact, create and communicate” [29].

### Competitive Cessation Internet Interventions (Promotion)

While smoking cessation websites are already available, many Internet users encounter frustrations on sites that are poorly designed. A study of 202 cessation websites found that only 46 provided actual cessation treatment, and only 5 of those received high rankings for usability based on evidence-based guidelines [30]. There were readability problems, no coverage of key components of recommended guidelines, and failure to provide interactive exercises and tailored messages [30]. Navigational issues, content layout, and information location are common problems [31]. Many require detailed profiles before entering the site. The two most frequently visited sites were owned by tobacco companies and perceived by respondents as unhelpful [32]. Seventy-two percent (13/18) of the most popular websites were for profit and several promoted unproven methods. The US Government’s Smokefree website [33] was ranked above average for helpfulness, but was strictly informational without interactive exercises. QuitNet provides some free information, but requires payment for use of the site for specialized services, which may be a barrier to participation [34]. The VA’s My HealtheVet website does provide some information about the benefits of quitting smoking, tips for quitting, and weight gain, but it is not a comprehensive intervention, and has limited interactivity and tailoring. The Department of Defense has a smoking cessation website that veterans can access [35]; however, we are not aware of any published studies on the efficacy of the website. A Google search for “quit smoking” did not reveal some of the most efficacious cessation websites. Hence, tobacco users who search the Internet for cessation assistance are unlikely to find high-quality, evidence-based treatment resources. Thus, the Tobacco Tactics website was developed in conjunction with a graphic design company, Allen Wayne, LTD (VA SHP 08-197).

## Methods

### Design

Both formative (process) and summative (outcome) evaluation were used across three small successive studies to develop and test the Tobacco Tactics website for veterans. Formative evaluation was conducted with a small group of veterans to “test run” the Tobacco Tactics website, obtain feedback, and then revise the website. Summative evaluation was conducted to determine if the website did what it was designed to do in two small pilot studies. Human studies approval was obtained from the VA Ann Arbor Healthcare System and the University of Michigan.

### Sample/Setting (Place)

The sample for all three studies consisted of veteran smokers and former smokers who had access to the Internet. The samples for the usability test and first pilot study were recruited at the VA Ann Arbor Healthcare System and were not necessarily smokers who were interested in quitting (usability testing included former smokers) while pilot study 2 consisted of all motivated smokers.

### Procedures (Promotion)

#### Usability Testing

Based on modules used in our prior studies, the Tobacco Tactics website was built with our website developers, Allen Wayne, LTD. First, the research team and website developers sat individually with 5 VA smokers and former smokers (employees or volunteers from Voluntary Services) for one hour as they navigated each successive webpage and requested feedback on: (1) ability to accomplish tasks, (2) ability to accomplish goals with skill and speed, (3) ability to operate the system, and (4) satisfaction with the website. Then, based on the feedback, the website was revised for pilot testing.

#### Pilot Study 1

Using the revised website, 9 veteran smokers (patients) who had computer access and were willing to test the website at home were recruited from the smoking shelter. Veterans were given a baseline survey and a 30-day follow-up survey to request information about their smoking habits and other covariates that can influence smoking.

#### Pilot Study 2

To further test the website among smokers motivated to quit who also received NRT, 18 veteran smokers who had computer access and were interested in quitting were recruited from Operating Engineers Locals 324 Training Center as part of a larger funded study. This is a registered randomized controlled study (clinicaltrials.gov NCT01124110). Veterans were randomized to the Tobacco Tactics website plus mailed NRT (ie, patch, gum, or lozenge) or the 1-800-QUIT-NOW telephone line, which also mailed NRT for those without insurance. The specific type of NRT was given based on the number of cigarettes the veteran smoked as well as his/her previous experience with NRT and personal preferences. Veterans were given a baseline survey and a 30-day follow-up survey to request information about their smoking habits and other covariates.

### Measures

#### Usability Testing

The questions for the usability testing were open-ended. Examples of the open-ended questions were: Why did you click on that? or Why did you skip that exercise?

#### Pilot Tests

The outcome of interest was self-reported seven-day point prevalence abstinence at 30-day follow-up. To be considered as having quit, the patient had to self-report on their 30-day follow-up survey that they had “not used any tobacco products, even a single puff of a cigarette, in the past 7 days.” Variables known to be associated with smoking and cessation were measured on the survey using previously validated tools including: (1) the Fagerström Test for Nicotine Dependence (FTND), (2) the Alcohol Use Disorders Identification Test (AUDIT), (3) the abbreviated Center for Epidemiologic Studies Depression Scale (CESD) [36], and (4) “self-rated health” [37]. Demographic variables included age, gender, race, education, marital status, and employment status. Comorbidity information was self-reported by patients.

### Description of Tobacco Tactics Website Intervention (Product)

#### General Description

The Tobacco Tactics website was developed based on prior work and is in keeping with guideline recommendations for treatment of tobacco addiction [38]. The content is written at an eighth grade reading level. Veterans can sign onto the website with a unique ID and user generated password. The Tobacco Tactics website [39] can currently be viewed with a test user login of “test” and password “testpass.”

#### Home Page

**Figure 1:**
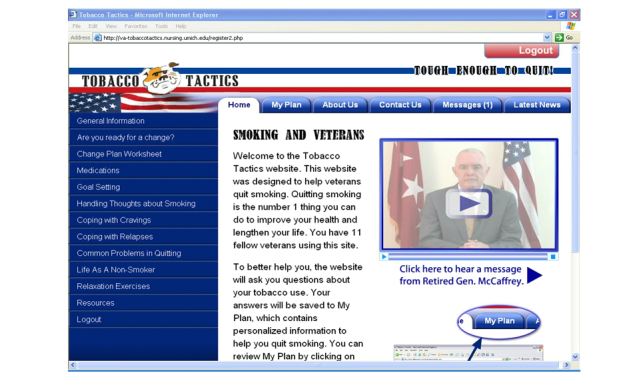
Sample Tobacco Tactics for veterans home page.

**Figure 2:**
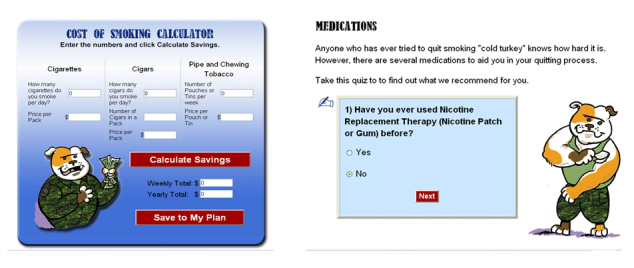
Sample Tobacco Tactics for veterans webpages.

As seen in Figure 1, the home page includes a video from General Barry McCaffrey who provides a compelling, emotionally charged message about the General’s personal story of smoking in the military and how he managed to quit. The left side of the home page has “buttons” that link to specific cessation modules including “General Information”, “Are You Ready for a Change?”, and the “Change Plan” button which has an interactive calculator that will assess the amount of money that can be saved from quitting (see Figure 2).

#### Medications

**Figure 3:**
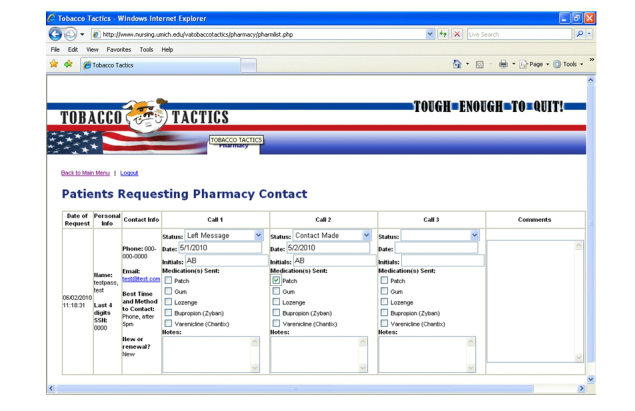
Sample Tobacco Tactics for veterans webpage for pharmacists.

Treatment links review the medications available to assist with cessation, including NRT (gum, patch, spray and lozenge), Zyban, and Chantix. An interactive exercise provides a tailored medication protocol and contraindications assessment (see Figure 2). On the Medications Tab, veterans can indicate whether they would like a pharmacist to contact them to provide medications and if so, they provide their name, email or phone number, and best time to be contacted. This information can be transmitted to the Pharmacy Administration webpage, which is part of the administrative section of the website, not accessible to patients. Pharmacists can routinely check the Pharmacy Administration webpage to see who has requested medications, review information on the website and the patients’ medical record, call, or email the patient to further assess, enter the medication request into the Computerized Patient Record System, and mail the prescription to the veteran. See Figure 3 for a screenshot of the Pharmacy Administration webpage.

#### Additional Buttons

Additional buttons on the left side of the home page include “Goal Setting”, “Handling Thoughts About Smoking”, “Coping With Cravings”, “Coping With Relapses”, “Common Problems in Quitting”, “Life As A Nonsmoker”, and buttons that provide relaxation exercises and resources. Tabs across the top of the home page include “About Us”, “Messages”, “Latest News”, and a tab for an e-community (chat room).

#### Interactivity and Tailoring

Extensive programming has resulted in the highly interactive exercises with tailored messages that save data to “My Plan” so users can monitor their progress over time. For example, a cost calculator depicts the amount of money that can be saved by quitting smoking. The e-community feature of the website consists of a three-day per week (one morning and two evenings), nurse-moderated forum that allows participants to conduct text-based discussions.

#### Graphics

The artwork and logo were developed using social marketing techniques based on prior work by Duffy [40], and lessons learned from advertising noting the use of animal cartoons (eg, Joe Camel, AFLAC duck, Smokey the Bear) as some of the most successful campaigns in attracting attention and selling products [41]. The development of the artwork is described in another paper [16] which describes how a graphic design firm, Allen Wayne, LTD, was hired to illustrate the logo and character for the VA Tobacco Tactics program. Initially, the firm developed thumb sketches of 4 logos and 10 variations of a character that would be portrayed doing different activities related to quitting smoking. Four rounds of formative evaluation were conducted by distributing surveys to veterans, visitors, and staff to elicit comments about the art work until there was high agreement from respondents that the logo, illustrations, and color were “good” or “excellent”.

#### Proactive/Warm Transfer Telephone Component and Email Reminders

Veterans who use the Tobacco Tactics website received a follow-up telephone call encouraging them to use the website and these calls also provided additional counseling. Proactive/warm transfer calls have been used to introduce patients to interventions in other studies [42] as well as business situations [43]. Veterans also received automated motivational emails to encourage them to use the website.

## Results

### Usability Testing

As a result of usability testing, more than 27 modifications were made including, but not limited to: (1) moving the user login box to the top of the page, (2) making the play button for the video more visible, (3) changing the font color of selected headings from blue so as not to confuse them with a URL link, (4) making the scrolling list of chemicals slower, (5) enlarging the calendar icon when setting a quit date, and (6) changing the wording to be more veteran-centric (eg, “Smoking causes more deaths than all Americans killed in World War I, World War II, and the Korean and Vietnam wars combined”).

### Pilot Study 1

Thirteen veteran smokers were approached to recruit 9 who had computer access and were willing to test the website at home. The 9 smokers were not necessarily motivated to quit nor were they provided any cessation medications. Sixty-seven percent (6/9) of veterans accessed the website with prompting by a follow-up telephone call. Overall, the average age was 48 years, 67% (6/9) were male, and 67% (6/9) were white. Sixty-seven percent (6/9) had some college education and 33% (3/9) had less than a high school degree or a high school diploma/GED. One-third of the sample was never married, 22% (2/9) were married, and the remainder were separated, widowed, or divorced. Forty-four percent (4/9) were unemployed. At 30-day follow-up, none of the 6 responders had quit, however, 1 had attempted to quit. Fifty percent (3/6) had cut down on the number of cigarettes, averaging a reduction of 7 cigarettes per day. Most found the website easy to read (n=5), enjoyable (n=4), and easy to navigate (n=4), rated the website highly on pictures, illustrations, and colors, and indicated that they would recommend the website to others (n=5).

### Pilot Study 2

To further pilot test the Tobacco Tactics website with veteran smokers who were motivated to quit, 18 veterans recruited from a population of Operating Engineers (heavy equipment operators) were randomized to the Tobacco Tactics website plus NRT versus the 1-800-QUIT-NOW telephone line. Groups were similar on baseline variables. At 30-day follow-up, 80% (8/10) intervention participants responded, while 63% (5/8) control condition responded. Using an intent-to-treat analysis (assuming non-responders in the control group were smokers and did not use NRT), Table 1 shows that seven-day point prevalence abstinence at 30-day follow-up was 40% (4/10) in the intervention group compared to 13% (1/8) in the control group. Seventy percent (7/10) of veterans in the intervention group used NRT compared to 13% (1/8) in the control group. The average participant accessed the website 2.5 times (range 1-6).

**Table 1:** Characteristics and smoking quit rates for Veteran Operating Engineers randomized to the Tobacco Tactics website intervention compared to control subjects (N=18).

		Baseline	
		Intervention (N=10)	Control (N=8)
		Mean (SD) or Frequency (%)	Mean (SD) or Frequency (%)
**Characteristics**			
	Age	45.6 (7.5)	46.3 (8.1)
	Male	10 (100%)	8 (100%)
	White	10 (100%)	6 (75%)
	High school or less than high school	7 (70%)	4 (50%)
	Marital status	7 (70%)	5 (71%)
	Nicotine dependence ^a^	5.0 (2.6)	3.9 (3.0)
	Baseline Problem Drinking (AUDIT) ^b^	8.9 (4.6)	10.6 (8.2)
	Baseline Probable Depression (CES-D) ^c^	12.1 (10.2)	10.3 (8.5)
**30-day follow-up**			
	Quit Rate	4 (40%)	1 (13%)
	NRT use	7 (70%)	1 (13%)

^a^The Fagerström Test for Nicotine Dependence ranges from 0-10; a score ≥ 8 is considered nicotine dependent.

^b^The AUDIT ranges from 0-40; a score ≥ 8 is considered problem drinking.

^c^The CES-D ranges from 0-60; a score of 16 or higher is indicative of significant or mild depressive symptomatology.

## Discussion

### Principal Findings

The two pilot studies showed that veterans were able to change their smoking behavior after exposure to the Tobacco Tactics website. The poor quit rate in the first pilot study was not particularly surprising in that none of the veterans were actively seeking cessation services and none received cessation medications. Nonetheless, 17% (1/6) of veterans had attempted to quit and the reduction in cigarettes per day was substantial for 50% (3/6) of the veterans. Moreover, participants rated the website highly. Albeit the study was underpowered, the seven-day point prevalence abstinence at 30-day follow-up in the second pilot study was more than three times as high in the intervention group compared to the control group as it included smokers motivated to quit who were also given NRT along with the website compared to the 1-800-QUIT-NOW telephone line where they may also have obtained NRT. These preliminary results are promising and suggest the need for wider-scale testing of the Tobacco Tactics website for veterans.

Most of the veterans who used the Tobacco Tactics website found it to be enjoyable, easy to read, easy to navigate, rated the website highly on pictures, illustrations, and colors, and indicated that they would recommend the website to others. This is likely due to the extensive usability testing that was conducted and subsequent revisions that were made. These revisions likely enhanced engagement and reduced navigational problems common on other websites [31].

Similar to other populations [44], about two-thirds of veterans have access to the Internet and were able to log on to the website with a proactive/warm transfer telephone call. Web-based cessation interventions, which have been shown to be efficacious [17-19], may allow smokers to quit on their own, reach veterans in rural areas, and may be more popular among younger, more Internet savvy OEF/OIF veterans, among whom smoking is especially pronounced [1,2]. The great advantage of the Tobacco Tactics website is that it can allow Veteran smokers to access cessation treatment, including medications, without ever leaving their home, reducing barriers in access to care. An additional advantage of the Tobacco Tactics website is that it could be broadly disseminated to veterans across the country extending the reach of cessation services in the VA. In addition to being convenient for veterans, it may in fact reduce face-to-face provider-veteran time.

**Figure 4:**
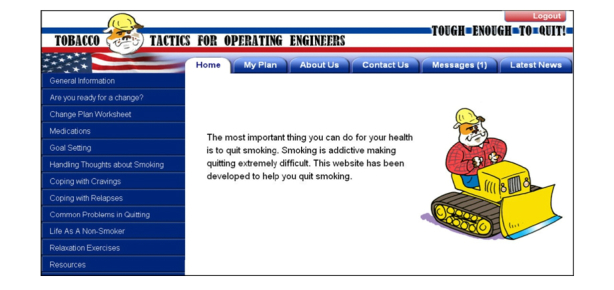
Sample Tobacco Tactics for Operating Engineers webpage.

The major barrier to implementation is that VA data security policies are such that there are several layers of “red tape” in order to house interactive websites on VA servers.  Thus, currently the Tobacco Tactics website is housed on a secure University of Michigan server.  For the moment, the VA is partnering with another website program [35] to service veterans. Nonetheless, with a grant from the Blue Cross Blue Shield of Michigan Foundation, we have redesigned the website for Operating Engineers (heavy equipment operators), which include a high proportion of veterans (20%) and are mostly men, similar to the veteran population.  In a funded National Institutes of Health (NIH) R21 for Exploratory Grants for Behavioral Research in Cancer Control (R21 CA152247-01) randomized control trial, the Tobacco Tactics website for Operating Engineers is being compared to the 1-800-QUIT-NOW state-supported quit line (see Figure 4).

### Limitations

The sample sizes for pilot studies 1 and 2 were small which may limit generalizability. Given that these were pilot studies, there was limited time for longer follow-up intervals as well as biochemical verification. Contact between participants and the research team may have influenced participants’ reports about the website. While data on the number of times the participants accessed the website was collected, no information was collected on the number of times they accessed particular exercises. The slightly higher differential drop out in pilot study 2 control subjects has implications for interpretation of the results, and may imply less engagement, as more intervention participants responded. Veterans assigned to the Tobacco Tactics website were given ready access to NRT while those assigned to the 1-800-QUIT-NOW group *may* have been able to obtain NRT.

### Conclusion

The preliminary results show that the Tobacco Tactics website has the potential to be an efficacious intervention for veterans trying to quit smoking. Moreover, the website has the potential to increase the reach of smoking cessation interventions to veterans who may have difficulty accessing services for younger OEF/OIF veterans who prefer the Internet. An efficacious tobacco cessation intervention that has the ability to reach a large number of veterans has the potential to increase smoking cessation rates and decrease morbidity and mortality in the VA.

## Abbreviations

NIH: National Institutes of Health
NRT: Nicotine Replacement Therapy
VA: Veterans Affairs
OEF: Operation Enduring Freedom
OIF: Operation Iraqi Freedom
AHRQ: Agency for Healthcare Research and Quality
FTND: Fagerström Test for Nicotine Dependence
AUDIT: Alcohol Use Disorders Identification Test
CESD: Center for Epidemiologic Studies Depression Scale
